# Re-measurement of agricultural carbon emission rates: Drivers, regional differences and dynamic evolution

**DOI:** 10.1371/journal.pone.0308496

**Published:** 2024-08-09

**Authors:** Yan Guo, Min Chen

**Affiliations:** 1 School of Economics, Ocean University of China, Laoshan District, Qingdao, Shandong, China; 2 Department of Economics, Sejong University, Seoul, South Korea; Shandong University of Technology, CHINA

## Abstract

This research paper introduces a novel approach by combining a Backpropagation (BP) neural network with a non-angular and non-radial directional distance function to construct a BPNN-DDF model. This innovative model evaluates, decomposes, and analyzes China’s agricultural sector’s carbon emission rate across nine key subregions between 2010 and 2021. The key findings of this study are that China’s agricultural carbon emission rate is decreasing, primarily due to technological advancements rather than technological efficiency. Subregions with robust economies and stable climates exhibit higher carbon emission efficiency, whereas those with underdeveloped economies, low agricultural technology, and volatile climates show relatively lower efficiency. The Dagum Gini coefficient analysis reveals a widening disparity in carbon emission rates among agricultural subregions, escalating from 0.174 in 2010 to 0.425 in 2021, indicating a growing gap between subregions that demands immediate attention. The kernel density distribution demonstrates an overall upward trend in China’s carbon emission efficiency but also highlights an increasing divergence among subregions, particularly between the South China Area, the Huang-Huai-Hai Plain, and other regions. Therefore, this paper posits that strategies focusing on technological progress, sustainable agricultural development, regional development initiatives, and addressing inter-subregional imbalances will be crucial pathways for China’s future low-carbon agricultural development.

## Introduction

Global food security is intricately tied to the sustainability and efficiency of agricultural green production. While driving economic prosperity, these practices contribute significantly to greenhouse gas (GHG) emissions—a staggering 23% of the global annual total [1]. Methane and nitrous oxide, primarily emitted through agricultural activities such as combustion, fertilizer use, and pesticide application, exacerbate climate change and threaten ecosystems [2], highlighting the critical need for a transformation towards low-carbon agriculture.

In this context, China emerges as a pivotal case study due to its rapid agricultural growth trajectory since the inception of economic reforms, with the total output value growing at an average annual rate of 12.63% in gross agricultural output value. It should be noted that the current unbalanced and insufficient development of Chinese agriculture is still prominent [3, 4], especially the sizeable regional gap in the green and low-carbon development of agriculture. This slows down the pace of global environmental governance and leads to persistent inequities in food security and resource utilization. Therefore, effective enhancement and balanced development of agricultural carbon emission efficiency can minimize environmental pollution and avoid overuse of resources, which is crucial for promoting sustainable agricultural development and realizing economic green transformation. However, the heterogeneous characteristics of inter-regional agricultural farming practices, soil conditions, climatic conditions, and resource endowments lead to spatial differences in carbon emission efficiencies across regions. Recognizing and studying such differences is crucial to formulating agricultural measures according to local conditions and giving full play to the practical benefits of green and low-carbon agricultural development. With this objective, our study innovatively adopts a BPNN-DDF model, merging artificial neural networks (BPNN) with a non-angular, non-radial directional distance function (DDF) under the umbrella of the general technology frontier concept. This advanced analytical tool enables a comprehensive evaluation, decomposition, and spatial analysis of carbon emission rate across nine key agricultural subregions in China from 2010 to 2021.

Given China’s dual role as the world’s most populous country and a significant agricultural producer, its agricultural zoning has been divided into nine regions by the Comprehensive Plan for Agricultural Zoning in China issued by the China Agricultural Zoning Committee. Northeast Plain (Heilongjiang, Jilin, Liaoning); Yunnan-Guizhou Plateau (Yunnan, Guangxi, Guizhou); Arid and Semi-Arid Regions of the North (Inner Mongolia, Xinjiang, Gansu, Ningxia, Qinghai, Tibet, Gansu, Ningxia); Arid and Semi-Arid Regions of the South (Inner Mongolia, Xinjiang, Gansu, Ningxia); Arid and Semi-Arid Regions of the North (Inner Mongolia, Xinjiang, Gansu, Ningxia), South China (Guangdong, Fujian, Hainan); Sichuan Basin and neighboring areas (Sichuan, Chongqing); Middle and Lower Yangtze River Plain (Jiangsu, Jiangxi, Hunan, Hubei, Anhui, Zhejiang, Shanghai); Qinghai-Tibet Plateau (Tibet, Yellow Huaihai Plain (Tianjin, Beijing, Shandong, Henan, Hebei). Focusing on China and exploring the characteristics of agricultural carbon emissions in these nine agricultural regions is strategically significant. Analyzing each agricultural subregion in detail makes it possible to reveal inter-regional differences and identify key areas for precise policy interventions. Therefore, the study period is selected from 2010 to 2021, when China’s economy and agriculture are undergoing a rapid transformation, to analyze the trends and dynamics of the carbon intensity of different agricultural regions in China.

This study aims to expose the geographical imbalance in the efficiency of agricultural carbon emissions and provide scientific support for promoting green and low-carbon agricultural development models and optimizing regional resource allocation. The core objective of this study is to enhance agriculture’s sustainability benefits and drive agriculture’s transformation towards eco-friendliness and low-carbonization to accelerate the realization of the overall sustainable development of agriculture. Through this exploration, we will provide data support and strategic guidance for policymakers and contribute Chinese solutions to global agricultural sustainability practices.

## Literature review

Agricultural carbon emissions, embodying the discharge of greenhouse gases from farming activities, necessitate meticulous measurement and evaluation to inform sustainable strategies. As a pivotal indicator, the agricultural carbon emission rate underscores the relationship between emissions and agriculture productivity, guiding the quest for eco-friendly growth [5]. A comprehensive indicator system further bolsters this pursuit, enabling in-depth assessments of green agricultural development [6, 7].

In the academic realm, research on agricultural carbon emissions has witnessed a surge, encompassing a diverse array of aspects, including analysis of the current state of agricultural carbon emissions [8, 9], carbon emission rate measurement [10, 11], identification of influencing factors [12], and exploration of emission reduction pathways [13]. While numerous studies focus on the direct measurement and analysis of agricultural carbon emissions, often incorporating economic indicators as auxiliary parameters, assessing the agricultural carbon emission rate transcends this limitation. It innovatively integrates non-desired outputs–carbon emissions–into the traditional agricultural productivity assessment framework, an adjustment that enriches the measurement criteria and significantly enhances the research findings’ realistic guiding value. Consequently, an increasing number of scholars have researched the agricultural carbon emission rate, primarily focusing on the following three aspects.

Firstly, the measurement method and characterization of the current agricultural carbon emission rate situation. Huang and Gao (2022) employed the DEA model to measure China’s agricultural carbon emission efficiency. They found that China’s agricultural carbon emission efficiency has improved during the survey period but remains low, with marked inter-provincial disparities. However, most regions exhibit a state of improvement [14]. Additionally, research scholars further explored its spatial effect, revealing convergence and evident spatial autocorrelation in agricultural carbon emission efficiency among provinces [14, 15]. Secondly, the mechanism of influence on the agricultural carbon emission rate is explored. Initial studies primarily focused on the decomposition of the agricultural carbon emission rate, revealing that its growth stems mainly from technological progress rather than efficiency improvement, albeit with slight differences across different regions [15, 16]. Subsequently, the focus shifted toward the exploration of factors influencing agricultural carbon emission efficiency, uncovering its susceptibility to various factors, such as mechanical inputs [17], labor migration [18], agricultural technological progress [19], and climatic environment [20, 21], among others. Thirdly, analyzing the spatio-temporal dynamics of the agricultural carbon emission rate has been a focal point. Researchers have explored regional differences [22, 23] and spatial and temporal changes [24], considering the differences in agricultural products, agricultural development, and regional economic conditions. For example, Zheng et al. (2024) integrated various approaches to analyzing China’s spatiotemporal dynamics of agricultural carbon emissions efficiency, demonstrating its evolution across different geographical locations over time [24].

The results of the existing research on agricultural carbon emission rate not only lay an essential theoretical and methodological foundation for this study but also highlight the shortcomings of the research on agricultural carbon emission rate. In the relevant agricultural carbon emission rate studies, parametric and non-parametric methods are mainly used to measure agricultural carbon emission productivity. Among them, non-parametric models have been widely used by scholars in agricultural carbon emission evaluation because they do not require pre-establishment of functional forms and a priori conditions (Molinos-Senante et al., 2016) [25] and can effectively avoid the subjectivity of parametric weighting (Dong et al., 2017) [26]. In the previous studies on agricultural carbon emission rate, most of them used the traditional data envelopment analysis (DEA) model or SBM-DEA model as the analytical method, mainly ignoring the effects of regional resource differences and negative outputs. They could not identify the contribution of different elements of inputs and outputs to the agricultural carbon emission rate. Therefore, based on the non-angle and non-radial DDF model proposed by Zhou et al. (2012) [27] and Shao et al. (2022) [28], this study innovatively introduces the BP neural network. It constructs the BPNN-DDF model to measure the carbon emission rate of China’s nine major agricultural subregions. Building upon this existing research, this paper makes several crucial advancements and refinements in the following four areas:

(1) We construct the BPNN-DDF efficiency measurement model by employing the non-angular and non-radial Directional Distance Function (DDF) of the overall technological frontier and integrating it with the Backpropagation Neural Network (BPNN). This approach also incorporates the Luenberger productivity index to address biases in setting the technological frontier and biased efficiency measurements. As a result, the accuracy of measuring China’s agricultural carbon emission rate across its 31 provinces from 2010 to 2021 is improved, with a focus on identifying the factors driving carbon emission efficiency.
(2) The meticulous decomposition of agricultural carbon emission efficiency in China’s nine subregions from the perspectives of input and output factors allows for precisely measuring the actual impact of changes in inputs and outputs on the agricultural carbon emission rate.
(3) Using the constructed BPNN-DDF efficiency measurement model and Dagum’s productivity indicator, we analyze the main factors contributing to enhancing agricultural carbon emission efficiency. This analysis provides valuable insights into the key drivers of efficiency improvements.
(4) Employing the Dagum Gini coefficient and kernel density estimation, we examine the spatial diversity of carbon emission efficiency within the nine subregions. Specifically, we focus on the evolving trends of disparities within and between subregions in the agricultural sector. This in-depth analysis will offer invaluable guidance for developing region-specific green growth strategies tailored to each subregion’s unique circumstances and challenges.

## Research methodology

### Measurement and decomposition model of agricultural carbon emission rate in China

#### Measurement model of agricultural carbon emission rate

Assume that each DMU receives N varieties of inputs and generates M varieties of anticipated output along with I varieties of unexpected output *b* = (*b*_1_,*b*_2_,⋯,*b*_*I*_). At each period *t* = 1,2,⋯,*T*, the output-input vector is represented by $\left(y_{k}^{t},b_{k}^{t},x_{k}^{t}\right)$. Referring to Zhou et al. (2012) [27], the subsequent explanation is regarded as applicable to DDFs that are not angular or radial, specifically about unforeseen results. The expression of the overall technology-based DDF, which is not angular or radial, can be achieved within the limitations of energy and the environment.

$$\vec{D^{0}}(x,y,b|g)=\sup\{w^{T}\beta :(y,b,x)+g\times \mathrm{diag}(\beta )\in P^{0}(x)\}$$ (1)

$w={\left(w_{m}^{y},w_{i}^{b},w_{n}^{x}\right)}^{T}$ is a vector of weights related to the quantities of output and input factors; *g* is a vector of directions, indicating that the desired directions of efficiency improvement are desired output expansion, undesired output, and input reduction; $\beta ={\left(\beta_{my},\beta_{ib},\beta_{nx}\right)}^{T}\geq 0$ is a Scaling Factor, whose values are the possible proportions of desired output expansion, undesired output, and input reduction. The DDF shown in Eq (1) measures the level of inefficiency of each input and output factor relative to the production frontier. Then, the DMU’s overall inefficiency level is measured using specific weights. The larger the value of the DDF, the lower the efficiency of its inputs and outputs, and vice versa, the higher the efficiency of its inputs and outputs. If the DDF value is 0, it is above the production frontier. The DDF for period t based on the overall technology frontier, i.e., the current agricultural carbon emission rate $\vec{D^{0}}(x^{t},y^{t},b^{t}|g^{t})$ can be obtained by solving the following linear programming model:

$$\vec{D^{0}}(x^{t},y^{t},b^{t}|g^{t})=\max\{\{\begin{array}{l} \max w_{m}^{y}\beta_{my}^{0,,t}+w_{i}^{b}\beta_{ib}^{0,t}+w_{n}^{x}\beta_{nx}^{0,t}\\ s.t.\sum_{k=1}^{K}z_{k}^{t}y_{km}^{t}\geq y_{m}^{t}+\beta_{my}^{0,t}g_{my}^{t},\forall m;\\ \sum_{k=1}^{K}z_{k}^{t}b_{ki}^{t}=b_{i}^{t}-\beta_{ib}^{0,t}g_{ib}^{t},\forall i;\\ \sum_{k=1}^{K}z_{k}^{t}x_{kn}^{t}\leq x_{n}^{t}-\beta_{nx}^{0,t}g_{nx}^{t},\forall n;z_{k}^{t}\geq 0 \end{array}\},t=1,\cdots ,T\}$$ (2)

#### BPNN-DDF model

Specifically for the agricultural carbon emission efficiency to be measured in this paper, this paper considers four input factors such as farmland inputs (F), labor inputs (L), machinery inputs (M), and resource inputs (R) (including resources such as fertilizers, diesel fuel, and films) in 31 provinces in China, the desired output Y (agricultural GDP), and the undesired output B (agricultural carbon emissions), at which time $X=(F,L,M,R),Y=(AGDP),B=(CO_{2})$. Referring to Zhou et al. (2012) [27] and Zhang et al. (2013) [29] to set the direction vector g=(Y,−B,−F,−L,−M,−R). To better reflect the differences in local inputs and outputs, unlike the proposal of Shao et al. (2022) [28], which assigns a weight of 1/3 to desired outputs, non-desired outputs, and input factors respectively, and distributes the weights equally according to the number of types of desired outputs, non-desired outputs, and input factors, the weight coefficients of each factor are calculated using BP neural network (BPNN). The BPNN can automatically determine the weights based on the input and output data, which makes the calculated value of agricultural carbon emission efficiency more accurate.

Based on the Luenberger productivity indicator form, the Agricultural Carbon Emission Efficiency (ACE) in period t+1 is defined as:

$$ACE=\vec{D^{0}}(x^{t},y^{t},b^{t}|g^{t})-\vec{D^{0}}(x^{t+1},y^{t+1},b^{t+1}|g^{t+1})$$ (3)

Since DDF measures the distance of DMUs from the production frontier (i.e., their inefficiency), ACE > 0 implies that the rate of agricultural carbon emissions has improved, and vice versa.

#### Decomposition of carbon emission efficiency in agriculture

According to the decomposition idea of the DEA efficiency measurement model proposed by Fujii et al. (2014) [30]. The decomposition of agricultural carbon emission efficiency can be obtained as a technical change indicator (TC) and efficiency change indicator (EC), along with the decomposition results considering input-output factors.


$$\begin{array}{l} TC=[\vec{D^{0}}(x^{t},y^{t},b^{t}|g^{t})-\vec{D^{t}}(x^{t},y^{t},b^{t}|g^{t})]\\ -[\vec{D^{0}}(x^{t+1},y^{t+1},b^{t+1}|g^{t+1})-\vec{D^{t+1}}(x^{t+1},y^{t+1},b^{t+1}|g^{t+1})] \end{array}$$ (4)

$$EC=\vec{D^{t}}(x^{t},y^{t},b^{t}|g^{t})-\vec{D^{t+1}}(x^{t+1},y^{t+1},b^{t+1}|g^{t+1})$$ (5)

$$\begin{array}{l} ACE=(w_{Y}\beta_{Y}^{0,t}+w_{B}\beta_{B}^{0,t}+w_{X}\beta_{X}^{0,t})-(w_{Y}\beta_{Y}^{0,t+1}+w_{B}\beta_{B}^{0,t+1}+w_{X}\beta_{X}^{0,t+1})\\ =(w_{Y}\beta_{Y}^{0,t}-w_{Y}\beta_{Y}^{0,t+1})+(w_{B}\beta_{B}^{0,t}-w_{B}\beta_{B}^{0,t+1})+(w_{X}\beta_{X}^{0,t}-w_{X}\beta_{X}^{0,t+1})\\ =ACE_{Y}+ACE_{B}+ACE_{X} \end{array}$$ (6)

### Spatial heterogeneity analysis of agricultural carbon emission rates

#### Dagum Gini coefficient

The Dagum Gini coefficient, a refinement of the traditional Gini index, offers a sophisticated statistical tool for assessing inequality in income or wealth and, as applied in this study, in carbon emission rates across different regions. Developed by Angelino DAGUM, this method enhances our understanding of disparity by decomposing the total inequality into three fundamental components: intra-subregional differences, inter-subregional differences, and a term accounting for the unevenness or ’density’ within those groups, known as hyper dispersion.

*Intra-subregional differences*. This component captures the heterogeneity of the nine agricultural regions. It measures how evenly or unevenly carbon emissions are distributed among provinces within the same region, reflecting variations due to local factors such as technology adoption, farming practices, and resource endowments.

*Inter-subregional differences*. This section focuses on regional disparities and quantifies how much the average carbon emission rates vary from one agricultural region to another. It highlights regional disparities influenced by broader economic, policy, and environmental differences across China.

*Hyper dispersion density*. A unique feature of the Dagum Gini coefficient, this term accounts for the overall spread or concentration of the distribution beyond what would be expected from a simple comparison of averages. It captures the degree of unevenness in the dataset, indicating whether the distribution is tightly clustered or widely dispersed, which could relate to outliers or extraordinarily high or low emission rates.

By employing this methodology, the study delves deeply into the multifaceted nature of carbon emission inequalities in China’s agriculture, enabling policymakers to discern whether mitigation efforts should target specific provinces with unusually high emissions (intra-subregional), address broad regional discrepancies (inter-subregional), or consider the influence of outliers and extreme values (hyper dispersion). Consequently, the insights garnered facilitate the formulation of tailored policies and strategies to foster green growth and promote sustainable agricultural practices across China’s diverse agricultural. According to Li et al. (2022) [31], the calculation formula is shown below:

$$G=\overset{h}{\underset{j=1}{\sum }}\overset{h}{\underset{d=1}{\sum }}\overset{n_{j}}{\underset{i=1}{\sum }}\overset{n_{d}}{\underset{r=1}{\sum }}|ACE_{ji}-ACE_{dr}|/2n^{2}\bar{u}$$ (7)

$$G_{w}=\overset{m}{\underset{j=1}{\sum }}G_{ij}p_{j}s_{j}$$ (8)

$$G_{nb}=\overset{m}{\underset{j=2}{\sum }}\overset{j‐1}{\underset{d=1}{\sum }}G_{jd}(p_{j}s_{d}+p_{d}s_{j})D_{jd}$$ (9)

$$G_{t}=\overset{m}{\underset{j=2}{\sum }}\overset{j‐1}{\underset{d=1}{\sum }}G_{jd}(p_{j}s_{d}+p_{d}s_{j})(1‐D_{jd})$$ (10)

Where: *h* denotes the number of agricultural subregions; *n*_*j*_(*n*_*d*_) denotes the number of provinces and municipalities in the subregion *j*(*d*); *ACE*_*ji*_(*ACE*_*dr*_) denotes the agricultural carbon emission rate of the provinces and municipalities in the subregion *j*(*d*); *n* denotes the number of provinces and municipalities; $\bar{u}$ denotes the mean value of the agricultural carbon emission rate of each province in China; *G*_*jj*_ denotes the Gini coefficient of the subregion *j*; *G*_*jd*_ denotes the Gini coefficient between the subregion *j* and the subregion *d*; *p*_*j*_ = *n*_*j*_/*n*, $s_{j}=n_{j}u_{j}/n\bar{u}$: *D*_*jd*_ measures the mutual influence of the agricultural carbon emission rate between the subregion *j* and the subregion *d*.

#### Kernel Density Estimation (KDE)

Kernel Density Estimation (KDE) is a technique for estimating the probability density function without making assumptions about its parameters. It estimates density by comparing distances between data points and using a smooth kernel function. Utilizing Kernel Density Analysis aids in elucidating the fluctuating pattern of carbon emission rates in China’s nine primary agricultural sub-regions, suggesting tailored approaches to enhance the carbon emission rate and foster agriculture’s environmentally friendly and sustainable growth. Its functional form is expressed as:

$$f(x)=\frac{1}{nh}\overset{n}{\underset{i=1}{\sum }}k(\frac{x_{i}-\bar{x}}{h})$$ (11)

In the above equation, *f*(*x*) is the density function, is the kernel function, *h* is the bandwidth, and *n* is the number of observations (i.e., the total number of provinces). *i* denotes individual provinces, *x*_*i*_ denotes independently and identically distributed observations, and is the mean. Since bandwidth (also known as width) is a critical parameter in the KDE, it determines the estimates’ smoothing degree. A bandwidth that is too small will result in an estimate that is too noisy or sharp, capturing too much of the sample specificity and ignoring the overall features; a bandwidth that is too large will make the estimate too smooth and may mask some essential features in the data. Determining the appropriate bandwidth is an essential issue in KDE. Commonly used methods include Silverman’s Rule of Thumb, cross-validation, interpolation, and adaptive kernel density estimation. Usually, a specific quantitative relationship between the sample size and the bandwidth needs to be satisfied, i.e., *h* is a function of *n* and the expression of its optimal solution is:

$$h={\left(\frac{4}{3n}\right)}^{\frac{1}{5}}\approx 1.06n^{-\frac{1}{5}}$$ (12)

In this paper, the Gaussian kernel is chosen for correlation analysis, and its functional expression form is:

$$k(x)=\frac{1}{\sqrt{2\pi }}\exp[-\frac{x^{2}}{2}]$$ (13)

Because the kernel function equation is complicated to determine precisely, nonparametric estimation often draws on graphical comparisons to show changes in the distribution of random variables clearly. In particular, the center of the density function shifts from side to side over time to reflect an increase or decrease in the level of the subject, with a shift to the right indicating an increase in level and a shift to the left indicating a decrease in level. There are two types of crests: "sharp and narrow" and "flat and wide". Typically, "sharp-narrow" peaks have higher peaks and a smaller range, which means that regional differences are decreasing, while "flat-broad" peaks are the opposite. If the curve shows more than one peak, it reveals that the overall observation is polarized. This paper employs Gaussian kernel density to study the changing patterns of carbon emission efficiency in nine primary agricultural subregions in China. Additionally, it investigates the spatial characteristics of the kernel density map, including its position, form, and expandability.

### Indicator selection and data sources

#### Selection of input-output indicators

Combined with the existing agricultural carbon emission rate research, this paper selects land, labor, and various agricultural production resources as input indicators in the agricultural production process. Table 1 displays the specific selection of indexes for agriculture’s land, labor, and resource inputs. The total sown area of crops determines land inputs, labor inputs are determined by the number of employees in the primary industry, and resource inputs mainly consist of fertilizers, pesticides, agricultural films, irrigation, machinery, diesel, and other agricultural production resources. The agricultural output indexes are measured by the gross agricultural output value calculated at the constant price in 2000. The non-desired output uses agricultural carbon emissions, referring to the calculation method of agricultural carbon emissions by Li et al. (2011) [32]. Based on the carbon sources of fertilizers, pesticides, diesel fuel, agricultural film, agricultural land, and irrigation, the corresponding carbon emission coefficients for calculation are 0.8956 kg/kg, 4.9341 kg/kg, 0.5927 kg/kg, 5.18 kg/kg, 312.6 kg/km^2^, and 19.8575 kg/hm^2^. Eq (14) illustrates the procedure for determining agricultural carbon emissions.


$$y=\overset{6}{\underset{i=1}{\sum }}y_{i}\times p_{i}$$ (14)

The above equation, *y* represents the total amount of carbon emission from agriculture; *y*_*i*_ represents the input amount of agricultural carbon source factor *i*; and *p*_*i*_ represents the carbon emission coefficients of *i* carbon source.

**Table 1:** Input-output indicators for agricultural carbon emission rates.

Criteria layer	Indicator layer	Sub-indicators	Indicator meaning	Unit
**Input**	Land Inputs	Farmland Inputs	Total sown area of crops	thousand hectares
	Labor inputs	Labor inputs	Number of workers in the primary sector at the end of the year	ten thousand
	Machinery Inputs	Agricultural Machinery Inputs	Total power of agricultural machinery	ten thousand kilowatts
	Resource inputs	Fertilizer Inputs	Fertilizer application(purified amount)	ten thousand ton
		Pesticide Inputs	Pesticide use	ten thousand ton
		Agricultural Film Inputs	Agricultural plastic film use	ton
		Irrigation Inputs	Effective irrigated area	thousand hectares
		Diesel inputs	Agricultural diesel fuel input	ten thousand ton
**Output**	Expected outputs	Agricultural output	Total agricultural output	billions
	Undesired outputs	Agricultural carbon emissions	Eq (14)	kg

### Data sources

To capture the variations in carbon emission rates among the nine main agricultural subregions in China and their evolving patterns, this study selects the period from 2010 to 2021 as the research timeframe. Considering data availability, a balanced panel dataset is constructed for the 31 provinces in mainland China during the specified period. The socio-economic indicators mentioned above are sourced from the China Statistical Yearbook for the years 2011–2022, along with the China Rural Statistical Yearbook, China Land and Resources Statistical Yearbook, and the statistical yearbooks of provinces (autonomous regions and municipalities under the central government). Table 2 displays the statistical information for the variables.

**Table 2:** Descriptive statistics of variables.

Variable Type	Variable Name	Mean	Standard	Min	Max
**Land Inputs**	Farmland Inputs	5314.955	3926.614	88.550	15009.810
**Labor inputs**	Labor inputs	853.243	643.199	33.380	2698.450
**Machinery Inputs**	Agricultural Machinery Inputs	183.662	145.683	4.220	716.090
**Resource inputs**	Fertilizer Inputs	5.290	4.168	0.080	16.490
	Pesticide Inputs	78874.700	66824.340	852	322965
	Agricultural Film Inputs	2122.043	1660.621	109.240	6212.760
	Irrigation Inputs	3303.340	2914.942	93.970	13353.020
	Diesel inputs	66.388	58.104	1.800	298.470
**Desired output**	Agricultural output	1498.787	1178.500	41.726	5533.379
**Undesirable output**	Agricultural carbon emissions	6106.752	4587.538	255.253	17290.980

## Measurement and decomposition results of agricultural carbon emission rate

### Trend of agricultural carbon emission rates

#### The overall trend of change

Using the BPNN-DDF approach, we assess the rate of carbon emissions from agriculture in all 31 provinces of mainland China between 2010 and 2021. To accurately assess the precision of the BPNN-DDF model in measuring carbon emission efficiency, this study introduces the super-efficient GML-SBM-DEA model with non-expected output as a comparison method. As shown in Table 3, the results of the comparative analysis show that the efficiency values estimated by the BPNN-DDF model exhibit higher volatility. This feature more fully reveals the significant differences in carbon emission efficiency between regions.

**Table 3:** Comparison of agricultural carbon efficiency results.

Year	BPNN-DDF	GML-SBM-DEA	Year	BPNN-DDF	GML-SBM-DEA
**2011**	1.1979	1.0657	**2017**	2.8288	1.0762
**2012**	1.1790	1.0420	**2018**	1.6041	1.0980
**2013**	1.7230	1.0686	**2019**	2.6045	1.0846
**2014**	0.9638	1.0553	**2020**	1.5475	1.0709
**2015**	1.5241	1.0806	**2021**	3.2527	1.1224
**2016**	1.7724	1.0989	-	-	-

**Fig 1:**
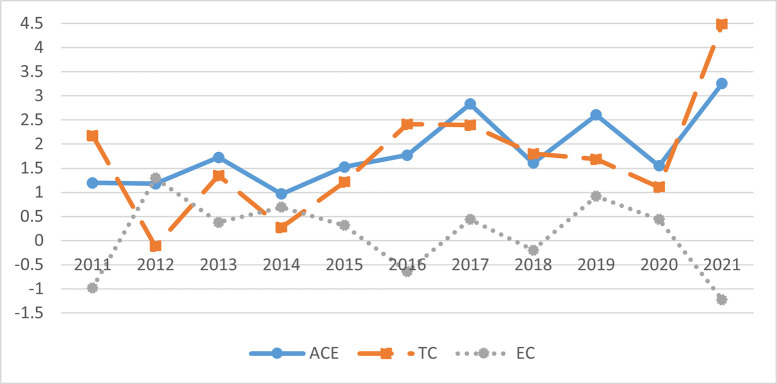
The overall trend of change in China’s agricultural carbon emission rate (%).

Additionally, we compute and break down the agricultural carbon emission efficiency (ACE) into two components: technical change (TC) and efficiency change (EC). This analysis allows us to evaluate the impact of both technical change and efficiency change on the overall efficiency of agricultural carbon emissions.TC represents the impact of the movement of the production frontier in each Chinese province, indicating the alteration in agricultural carbon emission efficiency caused by regional technological advancements. On the other hand, EC signifies the change in proximity of each Chinese province to the production frontier, reflecting the province’s progress in catching up with agricultural carbon emission efficiency. The specific results are shown in Fig 1.

Based on the data presented in Fig 1, it is clear that China has witnessed a consistent upward trend in agricultural carbon emission efficiency, exceeding a value of 1 from 2011 to 2021. A strong correlation is observed between the direction of change in agricultural carbon emission efficiency and technological progress, with the latter emerging as the primary catalyst for enhancing China’s agricultural carbon emission efficiency. The value of technology change significantly surpasses the value of efficiency change, underscoring the paramount importance of technological advancements in this context. Notably, between 2011 and 2016, there was a substantial shift in the technology indicator, which can be attributed to the implementation of energy conservation and emission reduction policies outlined in the 12th Five-Year Plan. The stringent environmental regulations played a pivotal role in stimulating innovation for energy savings and emission reductions across all Chinese provinces, thereby contributing to enhanced agricultural carbon emission efficiency. The examination of TC and EC trends reveals that before 2016, agricultural carbon emission efficiency was predominantly influenced by technical efficiency. However, from 2016 to 2021, the main driving factor shifted to changes in efficiency, as evidenced by the consistent dynamic fluctuations in agricultural carbon emission efficiency and efficiency changes. This shift could be because the technological frontier of agricultural production expanded significantly before 2016 due to the rapid growth of China’s agricultural economy. This expansion led to improvements in the efficiency of agricultural carbon emissions through technological innovations. Nevertheless, the swift advancement of the technological frontier made it challenging for many provinces to keep pace, resulting in fluctuating efficiency changes during this period. Consequently, efforts to enhance efficiency became increasingly crucial in maintaining the upward trajectory of agricultural carbon emission efficiency after 2016.

### The trend of carbon emission efficiency changes in nine major agricultural subregions

**Fig 2:**
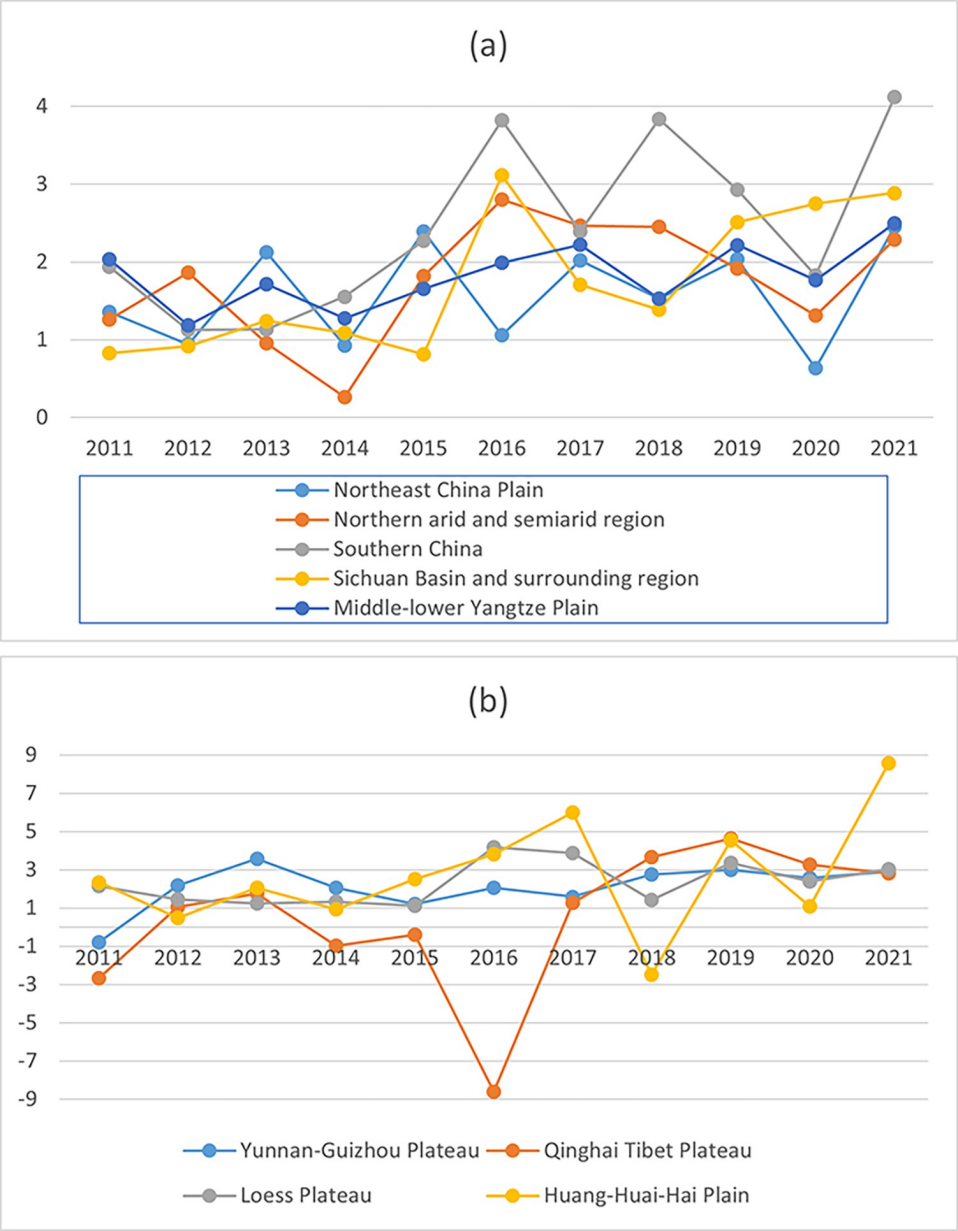
Trends in carbon emission efficiency in nine major agricultural subregions in China (%).

To clearly show the changes in agricultural carbon emission efficiency in each agricultural subregion, this study plotted the subregions in Fig 2(A) and 2(B) according to the degree of fluctuation in agricultural carbon emission efficiency. Fig 2 presents the average carbon emission efficiency change values for China’s nine major agricultural subregions, as measured by the BPNN-DDF model from 2010 to 2021. The analysis reveals that the mean carbon emission efficiency of these nine primary agricultural subregions exceeds 0, indicating a positive trend in their carbon emission efficiency. Notably, technological advancements emerge as the critical driver behind improving carbon emission efficiency across the nine primary agricultural subregions. As evidenced by Table 3, the mean value of technological change in these nine major subregions is higher than 0 and surpasses the mean value of efficiency change. This underscores the pivotal role that technological change plays in boosting carbon emission efficiency within China’s nine major agricultural subregions. In summary, the data presented in Fig 2 and Table 4 collectively demonstrate the significance of technological progress in enhancing the carbon emission efficiency of China’s nine primary agricultural subregions. These findings emphasize the need for continued investment in technological innovations and supportive policies to facilitate further improvements in carbon emission efficiency and promote sustainable agricultural practices across these subregions.

**Table 4:** Carbon emission efficiency of the nine major agricultural subregions in China (%).

**Division**	**TC**	**EC**	**ACE**
**Northeast China Plain**	0.31	1.27	1.59
**Yunnan-Guizhou Plateau**	0. 40	1.70	2.10
**Northern Arid and Semiarid Region**	0. 83	0. 94	1.76
**Southern China**	0.00	2.45	2.45
**Sichuan Basin and Surrounding Region**	0. 34	1.41	1.75
**Middle-lower Yangtze Plain**	-0.06	1.89	1.83
**Qinghai Tibet Plateau**	-2.77	3.30	0. 53
**Loess Plateau**	0. 68	1.65	2.32
**Huang-Huai-Hai Plain**	0. 95	1.76	2.72

Meanwhile, this study examines the pattern of carbon emission effectiveness in the nine primary agricultural subregions during the specified research period. Additionally, Fig 2 illustrates the carbon emission efficiency trend of these subregions in China using BPNN-DDF within the same research period. The results reveal that the carbon emission efficiency in most of China’s agricultural subregions is higher than one and primarily varies between 1 and 2. The Qinghai Tibet Plateau and the Huang-Huai-Hai Plain do not exhibit a noticeable upward or downward trend. This stability could be attributed to these regions’ unique environmental and socio-economic conditions.

Subregions with a stable agricultural environment, such as Southern China and the Huang-Huai-Hai Plain, have relatively high carbon emission efficiencies. This stability is likely due to consistent climatic conditions and fewer natural disasters, facilitating more efficient agricultural practices and lower carbon emissions. Conversely, areas characterized by complex agricultural development conditions, including the arid and semi-arid regions in the North and the Middle-lower Yangtze Plain, exhibit comparatively lower efficiency in carbon emissions. This can be attributed to harsher climatic conditions, water scarcity, and greater vulnerability to extreme weather events, hindering efficient agricultural practices and increasing carbon emissions.

Furthermore, regions with more advanced development levels, such as Southern China and the Huang-Huai-Hai Plain, tend to demonstrate relatively elevated agricultural carbon emission efficiency. This higher efficiency is likely due to better access to advanced agricultural technologies, improved infrastructure, and more robust economic support systems, which enable more efficient resource use and lower carbon emissions. In contrast, less developed regions, such as the Qinghai Tibet Plateau and the Northeast China Plain, exhibit comparatively lower carbon emission efficiency. This disparity can be attributed to limited access to modern agricultural technologies, inadequate infrastructure, and lower economic development levels, resulting in less efficient resource use and higher carbon emissions.

Overall, regional economic development and advancements in agricultural science and technology positively influence the efficiency of agricultural carbon emissions [33]. These factors enable regions to implement more efficient agricultural practices, thus reducing carbon emissions. Future policies should enhance technological adoption and infrastructure development in less-developed regions to improve carbon emission efficiency.

### Decomposition results of agricultural carbon emission efficiency

#### Decomposition of scale effect, environmental control effect, and comprehensive input effect

**Fig 3:**
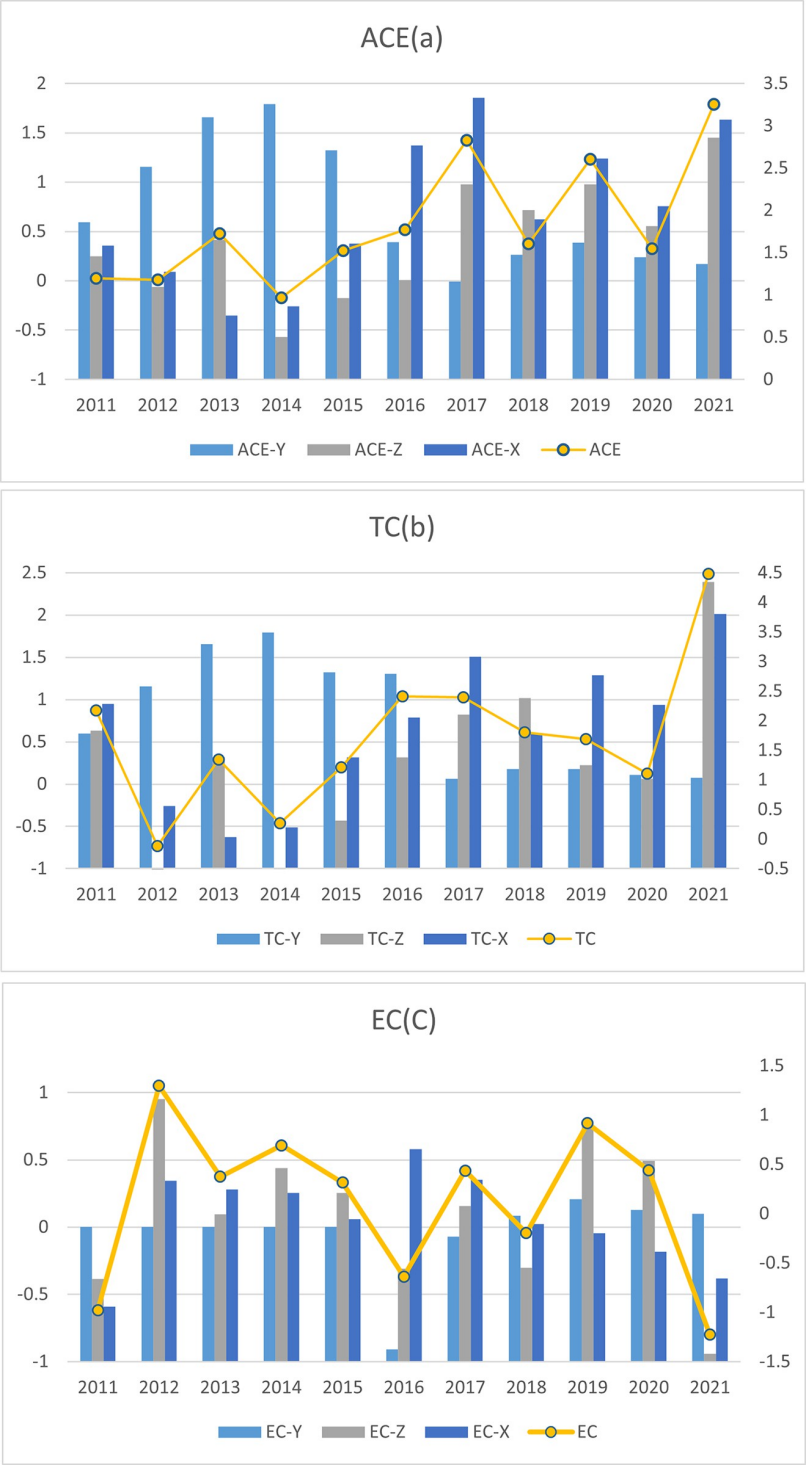
Decomposition of input-output effects of ACE (a), TC (b), and EC (c) (%) (%).

An analytical approach focusing on input and output factors is employed to effectively examine the efficiency of agricultural carbon emissions in China, drawing upon the foundational calculations established by a preceding model. This methodology facilitates a nuanced understanding of how each input-output factor distinctly contributes to technological advancements and productivity enhancements. The analysis, spanning a decade from 2011 to 2021, is succinctly encapsulated in Fig 3. This figure illustrates the influence of key factors on critical indicators, including the scale effect, the environmental control effect, and the comprehensive input effect. These indicators not only gauge the efficiency of agricultural carbon emissions but also provide insights into the progress of technical efficiency within the sector.

Before 2015, the growth of China’s agricultural economy did not significantly influence the enhancement of agricultural carbon emission efficiency. This observation becomes apparent when examining Fig 3(A)–3(C), where it is apparent that despite environmental constraints, increased agricultural production did not correspondingly improve the efficiency of agricultural carbon emissions. In contrast, technological advancements have been instrumental in enhancing this efficiency. Furthermore, the impact of environmental control has demonstrated a positive trajectory, particularly between 2015 and 2021. Technological advancements have consistently contributed to agricultural carbon emission efficiency growth during this period, counterbalancing any negative impacts from efficiency changes.

Like the environmental control effects, the aggregate input effects generally contribute positively. With the exceptions of 2013 and 2014, these inputs have maintained an economizing bias. However, when considering the technical and efficiency aspects, a trade-off emerges. On one hand, increased efficiency in factor utilization can lead to reduced consumption of various inputs. However, maintaining pace with these efficiency improvements becomes challenging as technology advances. This is evident in Fig 3(B), where efficiency changes shown in Fig 3(C) are minimally impactful in the face of negative technological changes before 2015. Conversely, changes in certain input factors can stimulate technological progress, but overly rapid advancements in the short term may result in efficiency gains that are difficult to sustain. This analysis suggests that technological advancements can facilitate some degree of input factor savings. To further enhance agricultural carbon emission efficiency, agricultural producers across all regions must embrace and apply advanced agricultural production technologies while continuously improving production factors’ efficiency.

#### Decomposition of the effects of the nine agricultural sub-regions

**Fig 4:**
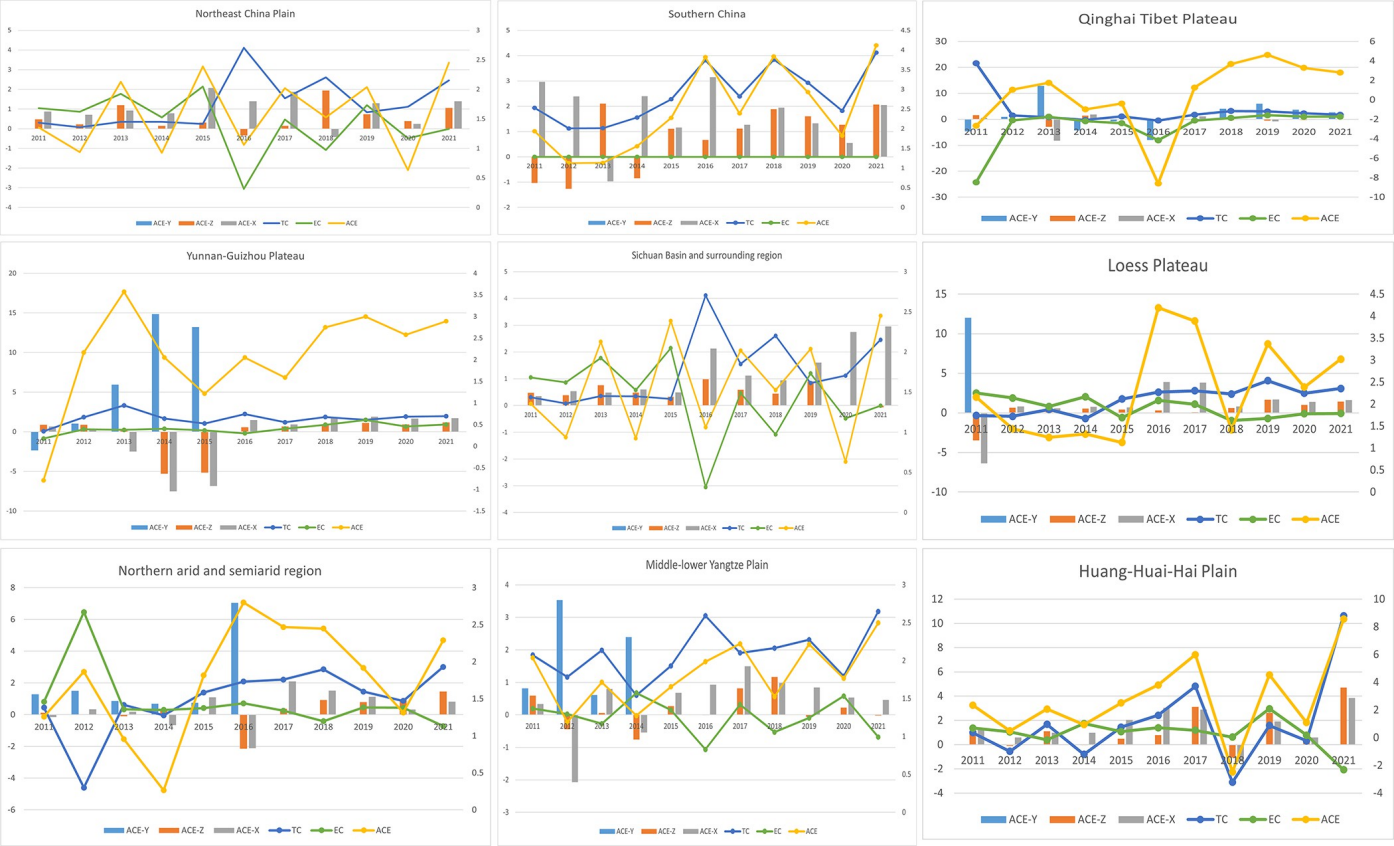
Input-output decomposition effects of carbon emission efficiency in nine agricultural subregions (%).

Fig 4 provides a detailed analysis of input and output factors, demonstrating the effects of scale, environmental control, and comprehensive input on carbon emission efficiency across China’s nine major agricultural regions. The analysis reveals that the scale effect on carbon emission efficiency in these regions is relatively minor, with all subregions exhibiting some degree of factor saving and environmental control. Notably, these effects have increased, particularly following China’s national policy for low-carbon agricultural development in 2015.

The variation in carbon emission efficiency among different agricultural subregions is attributable to their varied levels of agricultural technology and the implementation of environmental controls. Developed regions like Southern China and the Middle-lower Yangtze Plain rely predominantly on technological advancements to boost carbon emission efficiency. These regions benefit from access to cutting-edge agricultural technologies, improved infrastructure, and better resource management practices, collectively enhancing carbon efficiency.

In contrast, less developed areas, such as the Qinghai Tibet Plateau, depend more on efficiency improvements than technological advancements to enhance carbon efficiency. These regions often face challenges such as harsh environmental conditions, limited access to modern technology, and inadequate infrastructure, making it more difficult to achieve significant technological advancements. Therefore, they focus on optimizing existing practices and improving resource use efficiency to reduce carbon emissions.

A significant finding is that during 2011–2015, the influence of efficiency improvements was more pronounced than that of technological advancements. This period saw regions implementing better resource management practices and optimizing agricultural processes to improve carbon emission efficiency. However, the trend reversed post-2015, with the impact of technological changes gradually surpassing efficiency improvements. This shift underscores the pivotal role of technological advancements in driving the growth of China’s agricultural economy and enhancing the efficiency of carbon emissions in the sector. Introducing new technologies, such as precision agriculture, improved crop varieties, and advanced irrigation systems, has significantly contributed to this improvement.

Nonetheless, it is critical to recognize the substantial role of efficiency changes in improving carbon emission efficiency in specific agricultural subregions. In regions where technological adoption is slower, continuous improvements in agricultural practices, resource management, and environmental control remain essential for enhancing carbon efficiency. For example, implementing more efficient fertilization techniques, better pest control methods, and improved soil management practices can significantly reduce carbon emissions.

The study highlights the multifaceted approach needed to improve carbon emission efficiency in China’s agricultural sector. While technological advancements are crucial, ongoing efficiency improvements and effective environmental control measures are equally significant. Policymakers should promote technological innovation and adopt best practices across all regions to achieve sustainable agricultural development and reduced carbon emissions.

## Subregional differences and dynamic evolution of agricultural carbon emission rates in China

### Subregional differences in China’s agricultural carbon emission rate

#### Overall differences in subregions

**Fig 5:**
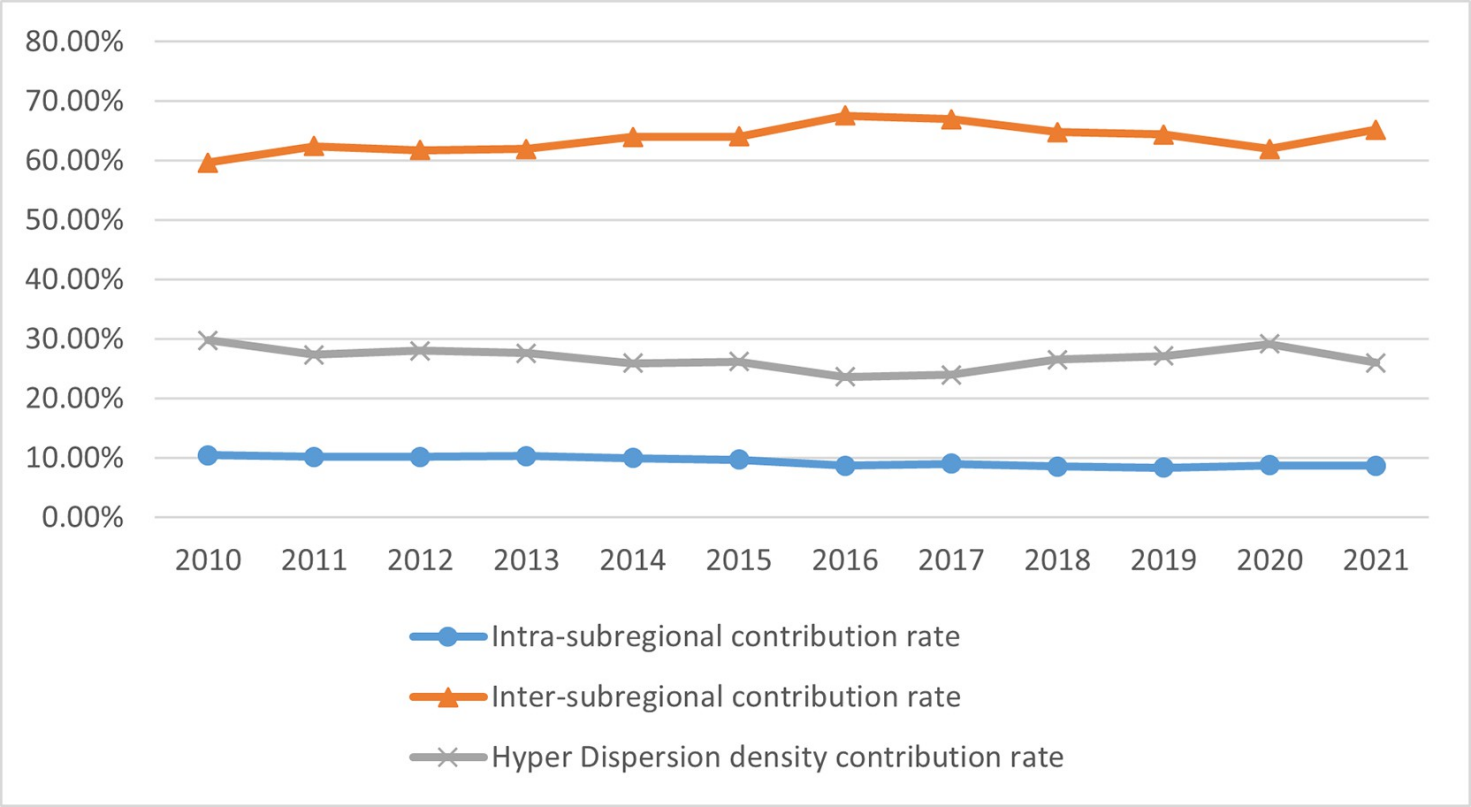
Trends in carbon emission efficiency differences among China’s nine key agricultural subregions.

Table 4 details the Dagum Gini coefficients for China’s nine main agricultural subregions between 2010 and 2021. Concurrently, Fig 5 graphically represents the evolving contribution rates of various factors that account for the disparities in carbon emission rates across these subregions. Table 4 indicates that the total Gini index for the country’s agricultural carbon emissions escalated from 0.174 to 0.425 over this period. This substantial increase indicates a growing divergence in carbon emission rates among the agricultural subregions.

A combined analysis of Table 5 and Fig 5 reveals that their differences are the primary drivers behind the variation in carbon emission rates across subregions. Notably, this factor follows a pattern of initial decline followed by an increase. Furthermore, the contribution rate of hyperintensity, which hovers around 25%, suggests that interactions between different subregions also play a significant role in shaping the disparities in agricultural carbon emission rates. In contrast, the impact of intra-zonal differences, although the least significant, has been on a downward trend. This comprehensive analysis underscores the complexity and dynamic nature of agricultural carbon emission rates across China’s diverse agricultural subregions.

**Table 5:** Gini coefficient and contribution to agricultural carbon emission rate in China, 2010–2021.

Year	Overall G	G_w_	G_nb_	G_t_	G_w_ Contribution Rate	G_nb_ Contribution Rate	G_t_ Contribution Rate
**2010**	0.174	0.018	0.104	0.052	10.49%	59.69%	29.82%
**2011**	0.192	0.020	0.120	0.053	10.18%	62.44%	27.38%
**2012**	0.194	0.020	0.120	0.054	10.19%	61.79%	28.02%
**2013**	0.194	0.020	0.120	0.054	10.35%	61.99%	27.66%
**2014**	0.199	0.020	0.128	0.052	9.99%	64.02%	25.99%
**2015**	0.213	0.021	0.136	0.056	9.72%	64.08%	26.20%
**2016**	0.241	0.021	0.163	0.057	8.74%	67.61%	23.66%
**2017**	0.289	0.026	0.194	0.069	9.04%	66.99%	23.97%
**2018**	0.297	0.026	0.193	0.079	8.61%	64.81%	26.57%
**2019**	0.325	0.027	0.209	0.088	8.39%	64.45%	27.16%
**2020**	0.346	0.030	0.214	0.101	8.80%	61.99%	29.22%
**2021**	0.425	0.037	0.277	0.111	8.75%	65.21%	26.04%

#### Intra-subregional differences

**Fig 6:**
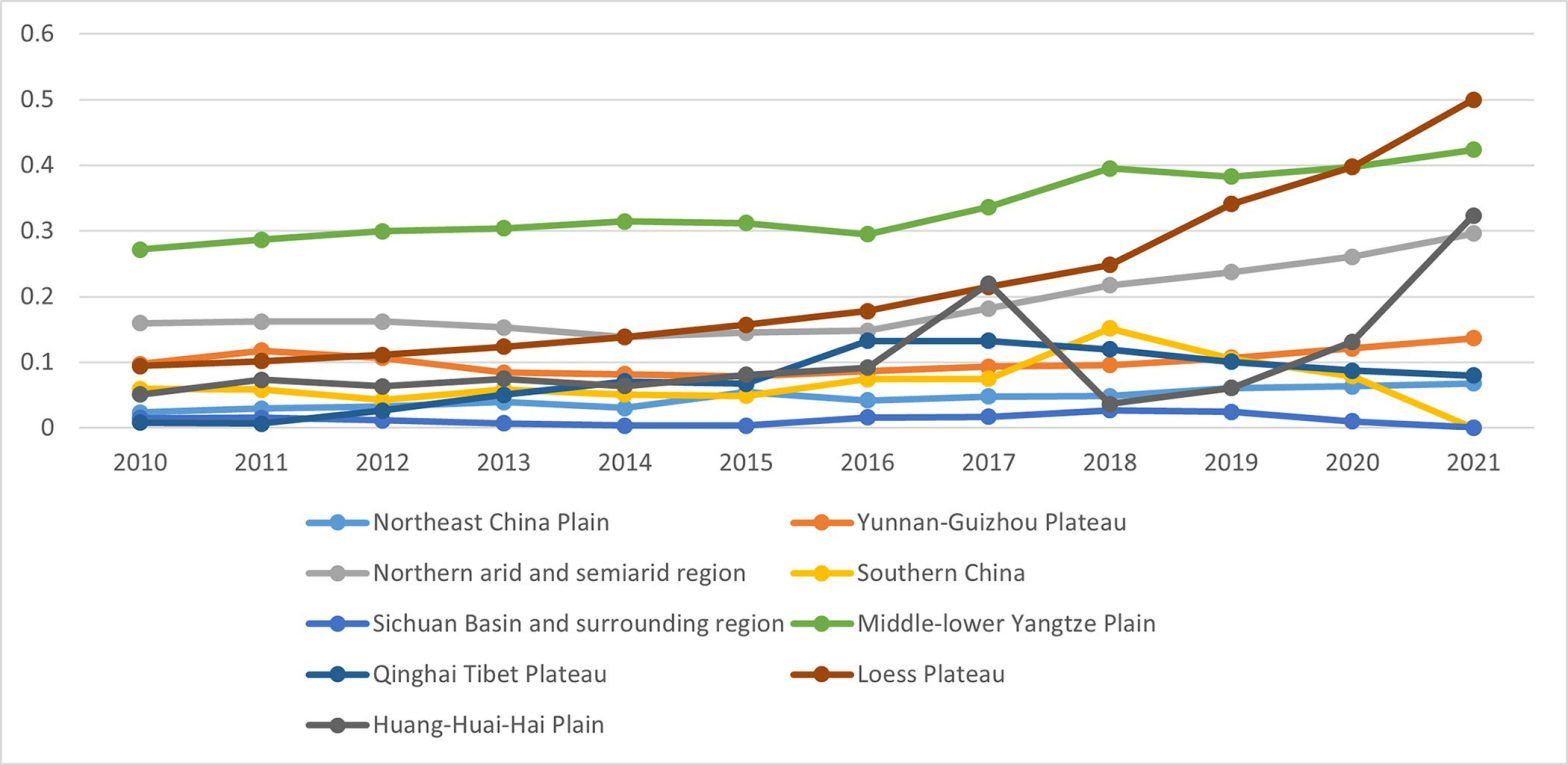
Intra-subregional carbon emission rate evolution in nine major agricultural subregions.

Fig 6 visually represents the evolving internal differences in carbon emission rates within China’s nine major agricultural subregions. Upon examining Fig 6, it becomes evident that specific subregions, namely the Middle-lower Yangtze Plain, the Northern Arid and Semiarid Region, the Loess Plateau, and the Huang-Huai-Hai Plain, exhibit significant internal variances. In contrast, subregions like the Yunnan-Guizhou Plateau and the Qinghai Tibet Plateau show relatively lower levels of internal variance. The slightest internal variances are observed in Southern China, the Sichuan Basin, and the surrounding region. The overall trend indicates a gradual increase in the internal differences among these subregions, with notable exceptions being Southern China, the Sichuan Basin, and the surrounding region, where the variance remains less pronounced. The underlying causes of these subregional variations in agricultural carbon emission rates are multifaceted. Economic development, geographic and environmental characteristics, and climatic conditions are key contributing factors.

Firstly, regions with higher levels of economic development, such as the Middle-lower Yangtze Plain and the Huang-Huai-Hai Plain, tend to have more advanced agricultural practices and technologies. This leads to more significant variability in carbon emission rates as some areas adopt new technologies faster than others. In contrast, less economically developed regions like the Qinghai Tibet Plateau and the Yunnan-Guizhou Plateau exhibit lower internal variance due to more uniform, traditional farming practices.

Moreover, geographic diversity significantly influences carbon emission rates. The Middle-lower Yangtze Plain and the Northern Arid and Semiarid Region have diverse topographies and soil types, leading to varied agricultural practices and carbon emission levels. Conversely, regions like Southern China and the Sichuan Basin have more homogeneous environmental conditions, resulting in more consistent carbon emission rates.

Additionally, climate plays a crucial role in agricultural productivity and carbon emissions. The Northern Arid and Semiarid Region and the Loess Plateau experience harsher climatic conditions, leading to significant agricultural practices and carbon emissions variability. In contrast, regions with more stable climates, such as Southern China, exhibit less internal variance in carbon emissions due to consistent agricultural practices.

In summary, the observed internal differences in carbon emission rates within China’s agricultural subregions are influenced by economic development, geographic and environmental characteristics, and climatic conditions. Understanding these underlying causes is crucial for developing targeted strategies to reduce carbon emissions and promote sustainable agricultural practices across all regions.

#### Inter-subregional differences

**Fig 7:**
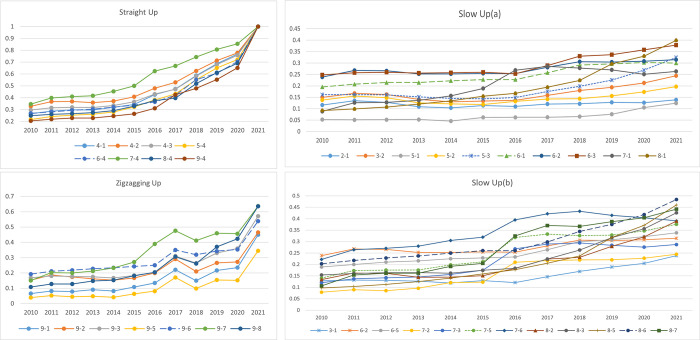
Evolution of carbon emission disparities in nine key agricultural subregions.

This paper categorizes the changes in inter-subregional agricultural variations into three patterns according to their dynamic trends: Straight Up, Zigzagging Up, and Slow Up. These trends are illustrated in Fig 7. In this case, because there are more slowly rising intervals, the subinterval differences are shown in two figures to show them clearly. Notably, Southern China encompasses subregions demonstrating a Straight-Up in inter-subregional differences, as detailed in Fig 7. This subregion’s superior agricultural production conditions, favorable climate, abundant natural resources, and advanced economic development contribute to its pronounced inter-subregional differences compared to other subregions. Consequently, it can be inferred that geographic location, climatic conditions, resource availability, and economic development level significantly influence subregional agricultural carbon emission efficiency.

Focusing on the subregions exhibiting a Zigzagging Up pattern, most are found within the Huang-Huai-Hai Plain. This trend is attributed to three main factors. First, the Huang-Huai-Hai Plain is located in China’s eastern monsoon climate zone, where climate change affects agricultural carbon emissions. Second, this area’s dense population and complex agricultural industry structure, characterized by extensive chemical fertilizers, pesticides, and other organic substances, can alter the subregion’s agricultural carbon emissions. Lastly, policy factors play a crucial role. In recent years, the government’s intensified efforts to protect the environment, promote renewable energy, advance technology, and restructure the agricultural sector have significantly influenced the Huang-Huai-Hai Plain, a subregion with a strong agricultural focus. Policies fostering low-carbon development in agriculture particularly impact this area.

Meanwhile, most other subregions exhibit a Slow-Up trend in inter-subregional differences. This differential change is likely closely associated with geographical development level, differences in geographic environments, agricultural production technology, and policy guidance. These elements collectively shape the patterns of change in agricultural carbon emission efficiency across the various subregions.

#### Dynamic evolution of carbon emission rates in nine major agricultural subregions in China

Based on measuring the subregional differences and sources of agricultural carbon emission rates in the nine subregions, this paper further adopts the KDE to examine the dynamic evolution characteristics of agricultural carbon emission rates in the country and the nine subregions.

#### Dynamic evolution of national agricultural carbon emission rate

**Fig 8:**
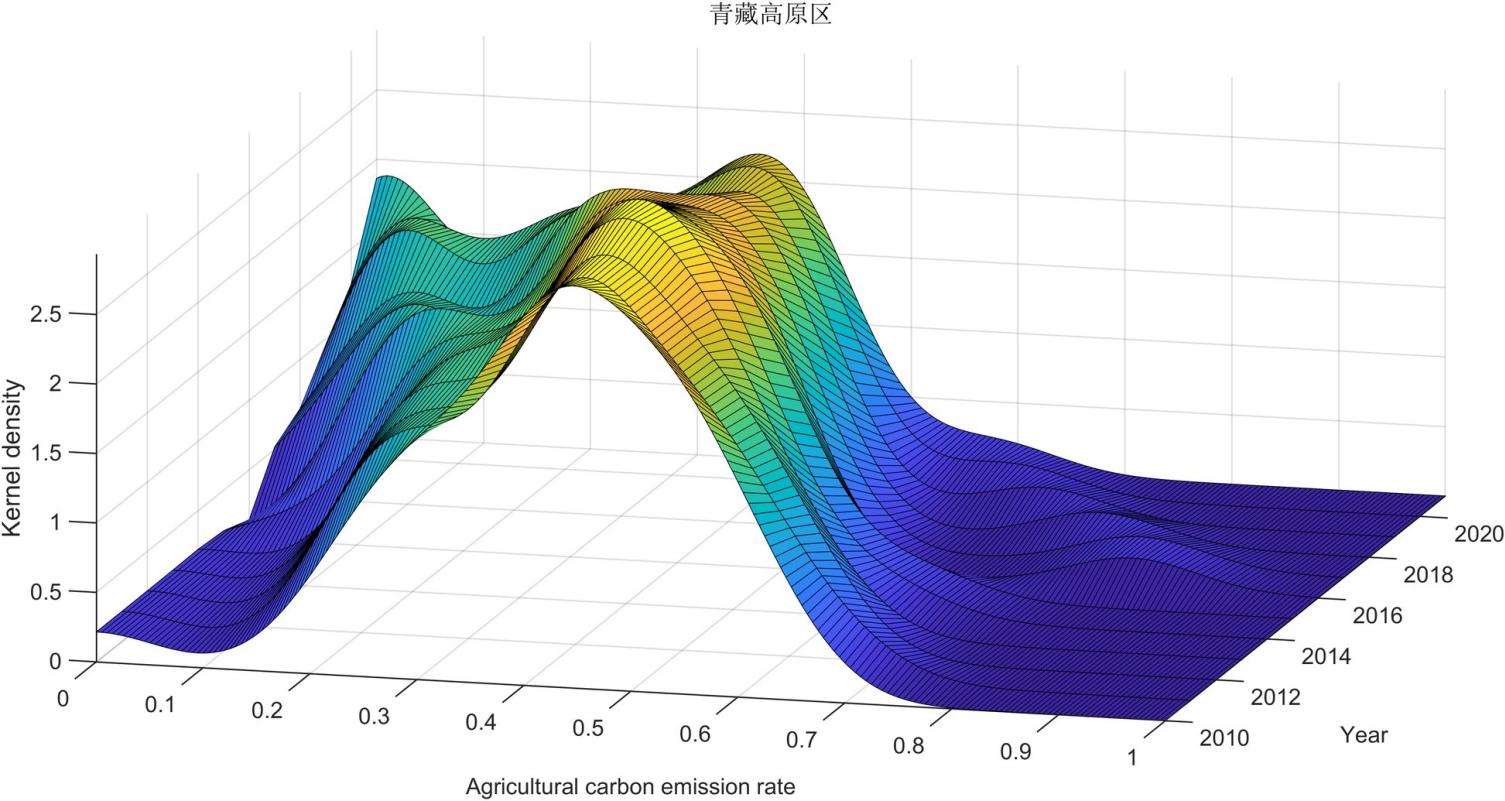
Dynamic evolution of national agricultural carbon emission rate.

Fig 8 illustrates the evolution of the kernel density curve for China’s national agricultural carbon emission rate. A notable observation from this figure is the shift of the central density function towards the left, indicating a gradual reduction in the agricultural carbon emission rate and a concomitant improvement in national carbon emission efficiency.

Analyzing the changes in the curve’s peaks, a distinct trend is discernible: the disparity in the national agricultural carbon emission rate initially increased from 2010 to 2013, then decreased from 2014 to 2018, and subsequently began to rise again. This pattern resonates with the findings from the earlier analysis on the national Gini coefficient, confirming a fluctuating trajectory of the disparities in agricultural carbon emission rates, characterized by an initial increase, followed by a decrease, and then an increase once more.

The national agricultural carbon emission rate also presents a ’double-peak’ distribution curve, indicative of a ’polarization’ phenomenon in China’s agricultural carbon emissions. As identified in the prior factor decomposition analysis, various factors, including environmental regulations and mechanical and labor inputs, influence the efficiency of these emissions.

During the 12th Five-Year Plan, the Chinese government fervently promoted eco-friendly development and implemented numerous energy conservation and emission reduction initiatives. These measures aim to advance technological capabilities and agricultural production techniques, leading to effective management and reduction of short-term agricultural carbon emissions. However, the rapid pace of urbanization and the increasing migration of rural workers have expanded agricultural output. This expansion, in turn, has caused a rise in carbon emissions in certain areas and variations in the rate of agricultural carbon release, highlighting the dynamic and complex nature of agricultural carbon emissions in China.

#### Dynamic evolution of carbon emission rates in nine agricultural subregions

**Fig 9:**
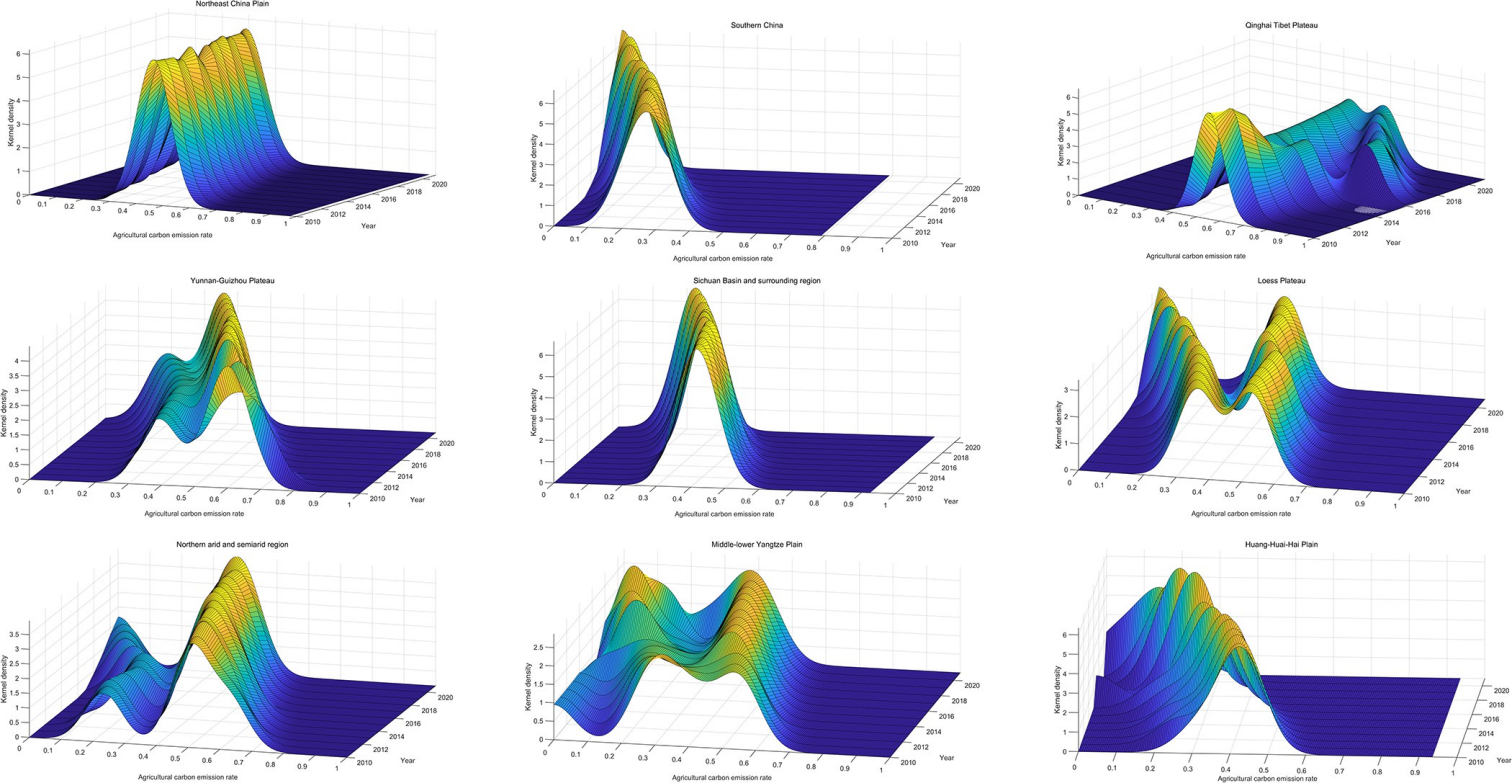
Dynamic evolution of carbon emission rates in nine agricultural subregions. Note: 1—Northeast China Plain; 2—Yunnan-Guizhou Plateau; 3—Northern Arid and Semi-arid Region; 4—Southern China; 5—Sichuan Basin and Surrounding Region; 6—Middle-lower Yangtze Plain; 7—Qinghai Tibet Plateau; 8—Loess Plateau; 9—Huang-Huai-Hai Plain.

Fig 9 showcases the distribution of carbon emission rates across nine primary agricultural subregions in China using kernel density curves. These patterns reflect the variations in carbon emission intensity across these subregions over different periods. An initial examination of the positional shifts in each area reveals that the kernel density functions for all nine agricultural subregions consistently move toward the left. This uniform shift suggests a declining agricultural carbon emission rate trend within each subregion, signifying improved carbon emission efficiency.

A closer look at each subregion’s peak alterations and shape characteristics reveals distinct patterns. The Northeast China Plain, Southern China, the Sichuan Basin, and nearby areas display a relatively uniform peak distribution, with the peak ascending and the right tail not extending significantly. This pattern indicates that spatial disparities within these subregions gradually decrease, leading to a more uniform pace of agricultural carbon emissions.

In contrast, the Northern arid and semiarid area, the Middle-lower Yangtze Plain, and the Loess Plateau show a more complex ’dual peaks’ distribution. The increasing distance between the two peaks and the elongation of the right tail suggest growing spatial disparities in carbon emission rates within these subregions each year. The Yunnan-Guizhou Plateau exhibits ’double peak’ characteristics. However, the distance between the peaks remains relatively stable, indicating more carbon emission rate stability than the previously mentioned three subregions.

Meanwhile, the Qinghai Tibet Plateau and the Huang-Huai-Hai Plain show more pronounced variations in the peaks and intervals of their carbon emission rates. These fluctuations suggest changing disparities among these subregions, with an upward trend. These observations are consistent with the earlier Gini coefficient analysis, further emphasizing variations in carbon emission efficiency across China’s nine primary agricultural subregions.

## Conclusion and policy implications

Utilizing the forefront of overall technology, this study integrates the BP neural network with a non-angular and non-radial directional distance function to develop the BPNN-DDF model. This innovative model is employed to comprehensively measure, decompose, and analyze the carbon emission rates in China’s nine major agricultural subregions from 2010 to 2021. The research delves into the subregional differences in agricultural carbon emission rates and their origins, employing the Dagum Gini coefficient. Additionally, the study leverages kernel density estimation to investigate the dynamic evolution characteristics of these subregional differences. From this thorough analysis, several key conclusions emerge:

(1) National Agricultural Carbon Emission Rate Trend. The findings align with previous studies [15, 16] that indicate an overall upward trend in China’s national agricultural carbon emission rate, predominantly driven by technological progress rather than improvements in technological efficiency.
(2) Inter-Subregional Efficiency Differences. The pronounced disparity in carbon emission efficiency among the nine subregions is consistent with the findings of Zhang et al. (2022) [22], who observed that subregions with advanced economies, higher levels of agricultural technology, and stable climatic conditions demonstrate greater efficiency in agricultural carbon emissions compared to less developed subregions like the Qinghai-Tibet Plateau. This highlights the influence of regional economic development, agricultural sophistication, and climatic conditions on carbon emission efficiency.
(3) Decomposition of Influencing Factors. The study’s findings, indicating a more substantial contribution of the environmental control effect and the comprehensive input effect to agricultural carbon emission efficiency than the scale effect, align with the observations of Yang et al. (2022) [34].
(4) Spatial Heterogeneity Analysis Using Dagum Gini Coefficient. The analysis reveals that spatial differences in carbon emission efficiency across the nine subregions are expanding. The primary drivers of these expanding disparities are inter-subregional differences, likely related to factors such as regional economic development, geographic location, climatic environment, and agricultural policies, consistent with the findings of Chen et al.(2019) [35] and Shan et al.(2022) [36].
(5) Kernel Density Estimation Findings. The observed national average decline in carbon emission rate, coupled with increasing differences in emissions between subregions and the "bimodal" distribution polarization in some regions. This means that, despite the overall development of China’s agriculture in the direction of decarbonization, there is still an urgent need to take targeted measures at the regional level to narrow the gap in the rate of agricultural carbon emissions between different regions and to promote balanced and sustainable development on a national scale.

Drawing from the insights gained, this paper proposes the following policy recommendations to enhance agricultural carbon emission efficiency:

(1) Strengthen technological innovation and promotion: actively promote the construction of an agricultural science and technology innovation system, increase the research and development and transformation of new, low-carbon, and high-efficiency agricultural technologies, especially in economically underdeveloped regions with relatively weak agricultural science and technology base, formulate targeted support policies, and accelerate the popularization of the application of advanced technologies through special funds, technological training, and demonstration projects, to ensure the effectiveness and sustainability of the transfer of technologies: effectiveness and sustainability.
(2) Refinement of regional differentiation strategies: Fully consider the ecological and environmental characteristics, differences in resource endowments, and the structural characteristics of agricultural production in the nine major agricultural subregions, and formulate and implement refined green development policies according to local conditions. Provide targeted support to lagging areas, including financial subsidies, technical support, market guidance, and other diversified means, to encourage them to change the traditional agricultural model and actively transform to modern agriculture that is environmentally friendly, resource-saving, and carbon emission inefficient.
(3) Optimize the allocation of agricultural machinery and equipment and labor resources: Vigorously promote the process of agricultural mechanization, especially in regions with conditions for large-scale mechanization, accelerate the pace of elimination and renewal of old agricultural machinery, and introduce energy-saving and highly efficient modernized agricultural machinery and equipment, to effectively reduce the problems of high energy consumption and carbon emissions caused by the traditional farming methods. At the same time, in labor-intensive agricultural areas, the reform of farmers’ education and skills training will be combined to improve farmers’ knowledge and practical ability in low-carbon agricultural production and energy-saving and emission-reduction technologies to cultivate a new generation of green farmers.
(4) Policy coordination and linkage and construction of long-term mechanism: Incorporate the efficiency of agricultural carbon emissions into the core indicator system of local economic and social development planning to ensure the synergy of the multiple objectives of economic growth, environmental protection, and sustainable development of agriculture, and to prevent the phenomenon of simply pursuing economic growth at the expense of environmental benefits. Establishing a long-term stable policy framework for green agricultural development, linking up and down the climate governance goals of governments at all levels, and gradually reducing the spatial variability of agricultural carbon emission efficiency among different regions through regulatory and institutional innovations, incentive mechanism design, and other measures, to realize the green and low-carbon transformation of agricultural production on a national scale.

## Supporting information

S1 Data (XLSX)
